# Localised anthropogenic wake generates a predictable foraging hotspot for top predators

**DOI:** 10.1038/s42003-019-0364-z

**Published:** 2019-04-04

**Authors:** Lilian Lieber, W. Alex M. Nimmo-Smith, James J. Waggitt, Louise Kregting

**Affiliations:** 1 School of Natural and Built Environment, Queen’s University Marine Laboratory, 12-13 The Strand, Portaferry, BT22 1PF Northern Ireland UK; 2 Marine Institute, University of Plymouth, Drake Circus, Plymouth, PL4 8AA England UK; 3 School of Ocean Sciences, Bangor University, Menai Bridge, Anglesey, LL59 5AB Wales UK

## Abstract

With rapid expansion of offshore renewables, a broader perspective on their ecological implications is timely to predict marine predator responses to environmental change. Strong currents interacting with man-made structures can generate complex three-dimensional wakes that can make prey more accessible. Whether localised wakes from man-made structures can generate predictable foraging hotspots for top predators is unknown. Here we address this question by quantifying the relative use of an anthropogenically-generated wake by surface foraging seabirds, verified using drone transects and hydroacoustics. We show that the wake of a tidal energy structure promotes a localised and persistent foraging hotspot, with seabird numbers greatly exceeding those at adjacent natural wake features. The wake mixes material throughout the water column, potentially acting like a prey conveyer belt. Our findings highlight the importance of identifying the physical scales and mechanisms underlying predator hotspot formation when assessing the ecological consequences of installing or removing anthropogenic structures.

## Abstract (web summary)

Lilian Lieber et al. examined seabird foraging around natural and man-made wakes, finding that wake from a turbine structure generates a more intense and predictable foraging hotspot for seabirds. This shows the importance of changes in physical forcing to top predators when installing or removing offshore structures.

## Introduction

In an era of intense marine urbanisation^1^, understanding scale-dependent physical forcing can help predict how marine predators may respond to environmental change. Predators rely on a multitude of physical processes, which dynamically influence foraging behaviour^2,3^ and success^4^. In the open ocean, predator foraging has been associated with mesoscale (10−100 km) physical features, such as fronts and eddies^5–7^. However, even fine- ( < 1 km, e.g., internal waves^3^) or local- (10−100 m, e.g., island wakes^8^) scale physical features may create small-scale predator hotspots^9,10^. The importance of these fine and local-scale physical processes is heightened in seabirds restricted to shallow plunge diving techniques, such as gulls and terns, where prey availability near the sea surface governs foraging site selection^11–13^. Consequently, tern species (*Sternidae*) tend to focus their foraging activity in areas of bathymetry-generated turbulence or shallow upwellings that consistently make prey available near the surface^11,12,14,15^. Such physically enhanced prey availability and its predictability seem to determine seabird foraging habitat rather than prey density alone^12,16–20^. Therefore, the identification of local flow processes interacting with bathymetric features (natural or man-made) can improve our understanding of the physical mechanisms promoting foraging hotspot formation and persistence in dynamic coastal systems^21^.

The periodic emergence of tidally driven bathymetry-induced turbulence, shallow upwellings or more ephemeral turbulent structures such as boils—circular regions of local upwelling^22^—are characteristic of strongly tidal seas. The introduction of anthropogenic structures into such dynamic environments adds further complexity to local flow processes, potentially triggering ecological implications^23^. Man-made structures modify local hydrodynamics^24^, including flow velocities^25^ and wake effects^26–28^. Further, a von Kármán vortex street^29^, characterised by distinct and repeatable eddy trajectories, may occur in the wake of embedded structures when placed in strong, near-laminar flows^30^. While fish may exploit the lee of a structure as a flow refuge^31^ or use small-scale vortices (e.g., < 1:1 ratio of vortex to fish size) to Kármán gait^32^, an extreme downstream wake with eddy vortices of sufficient size and vorticity^33^ can vertically displace or overturn fish in fast, unsteady flows^31,34–36^, potentially making them accessible to surface-foraging predators.

**Fig. 1:**
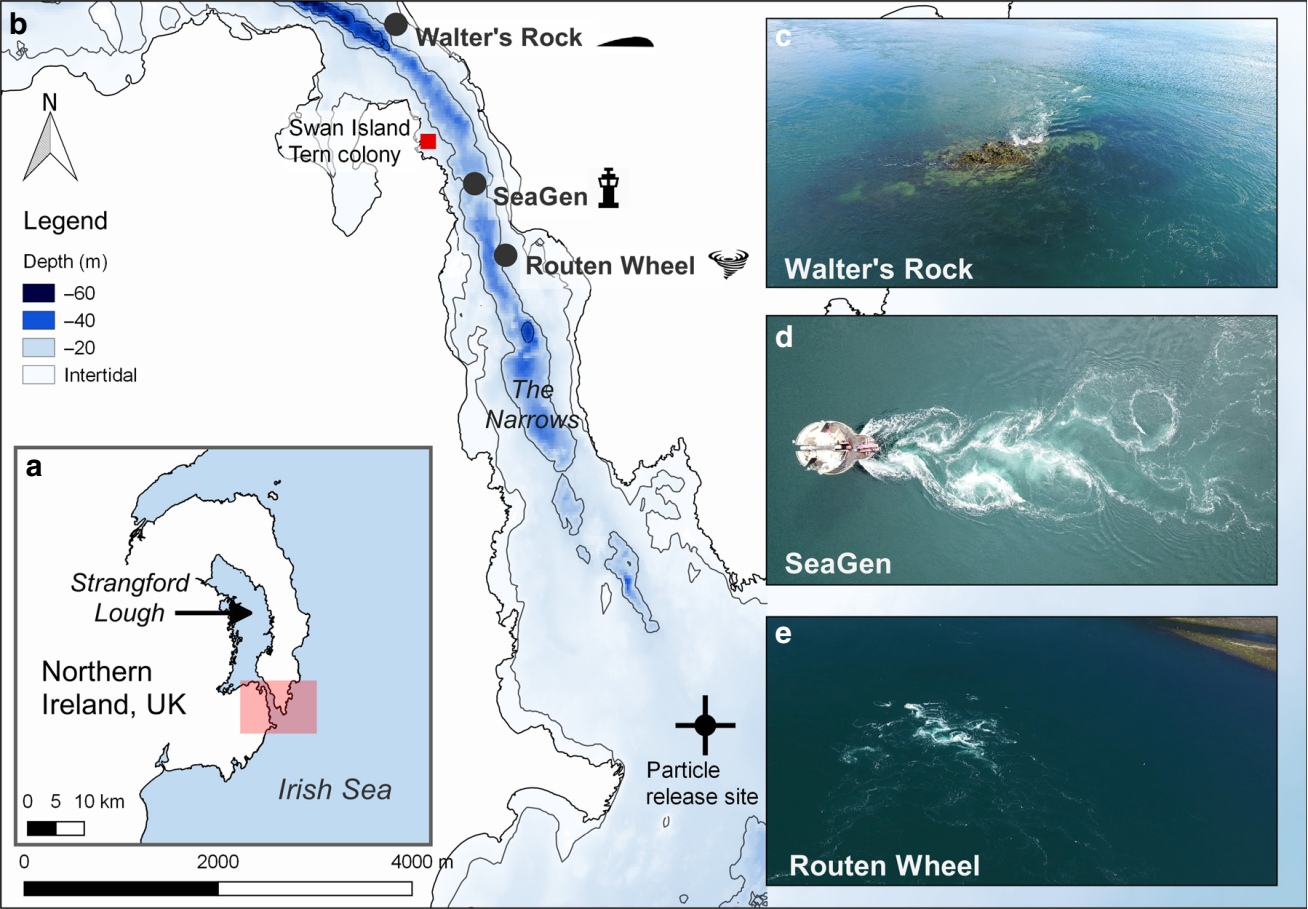
Location of wake features in the Narrows tidal channel situated in Strangford Lough, Northern Ireland, UK. **a** Overview map showing the study area within the Narrows, highlighted by a red box. **b** Location of wake features in the Narrows. **c**–**e** Insets showing the turbulent structures associated with each wake feature. Note: particle release site indicates the release of passive particles (as a proxy for prey organisms) from the Irish Sea during flood tide within a hydrodynamic model. OSNI data was reproduced from Land and Property Services data with the permission of the Controller of Her Majesty’s Stationery Office, © Crown copyright and database rights MOU203. Bathymetry: © Crown Copyright/SeaZone Solutions. All Rights Reserved. Licence No. 052006.001 31st July 2011. Not to be Used for Navigation

We hypothesised that a vortex street attributable to a man-made structure could present an as yet unexplored mechanism for localised predator hotspot formation. Here, we investigate whether a localised ( < 1 km) anthropogenically generated wake can present a reliable foraging location for surface-feeding seabirds (*Sternidae*), comparable to those at adjacent natural wake features. SeaGen, the world’s first grid-connected tidal energy turbine, currently being decommissioned, produces a wake with vortex shedding approaching a von Kármán vortex street^30^. The device consisted of a monopile structure (3 m diameter) attached to a quadropod foundation fixed on the seabed (water depth about 25 m) with a 27 m long crossbeam supporting the original rotors on either side of the tower 15 m above the seabed. During this study, the rotors had already been removed, however, the monopile itself contributes considerably to the vortex shedding in the downstream wake as shown through large eddy simulations^30^. SeaGen is situated in a dynamic tidal channel (‘‘the Narrows’’) in Strangford Lough, Northern Ireland, in proximity to colonies of summer-breeding tern species (*Sterna hirundo, S. sandvicensis, S. paradisaea*). The channel also provides diverse foraging opportunities with natural wake features commonly used by terns, therefore presenting a suitable study system. Two neighbouring extreme natural wake features, an island (Walter’s Rock) and a whirlpool structure (Routen Wheel), within the channel were selected to compare the terns’ use of the natural wakes with the man-made wake (Fig. 1). Our findings show that among all three wake features investigated, the flood wake associated with the man-made structure promotes the most persistent and intense foraging aggregations of terns. We further provide evidence that foraging over the wake is highly localised, highlighting the importance and ecological implications of localised physical forcing around man-made structures.

## Results

### Tern foraging patterns vary among wake features

The number of terns foraging at each wake feature was assessed using vantage point surveys (July–August 2018) with observations covering different tidal states (ebb versus flood, spring versus neap), recording variations in tern abundance across hydrodynamic conditions. The occurrence of conspicuous topographic and anthropogenic landmarks allowed the construction of plots with approximately the same area, with calculations based on bearings and distances from the vantage point. For SeaGen, observations were spatially divided into North (area of flood tide wake) and South (area of ebb tide wake) of the foundation, respectively. While the physical structure of SeaGen’s wake does not differ between the flood and ebb tide, the spatial separation was needed to ensure equal spatial extent per site. Further, it helped to assess whether terns were solely attracted to the environmental cue of turbulence (ecological trap^37^) or if aggregations were coupled to the ebb-flood tidal cycle.

**Fig. 2:**
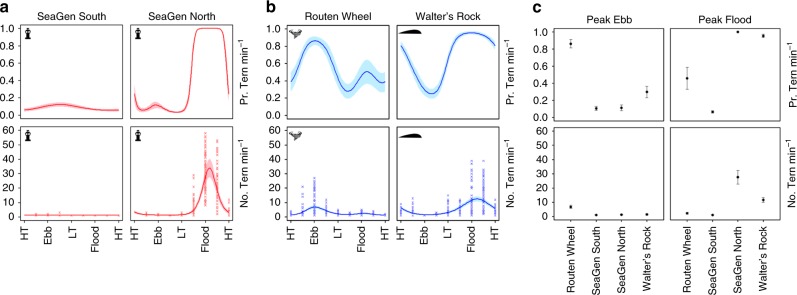
Tern counts over tidal state at each wake feature. **a**, **b** Mean ± SE variations in the predicted probability of encountering terns and the number of terns if encountered per minute across tidal states around SeaGen North and South (**a**), the Routen Wheel and Walter’s Rock (**b**) wake features, respectively. Crosses indicate the recorded number of terns if encountered binned into periods representing eight different states (1 h 20 min) of the ebb-flood cycle. HT = high tide, LT = low tide. **c** Mean ± SE variations in the predicted probability of encountering terns and the number of terns if encountered per minute across tidal states and locations. Tidal states represent peak current speeds in ebb and flood directions. All predictions (**a**–**c**) were made using model parameters from a general-additive mixed effect model (GAMM) with significance in both probabilities and numbers across tidal states shown in Table 1

Tidal coupling was evident with the highest probability of encountering terns at SeaGen North and Walter’s Rock during flood tides, and Routen Wheel during ebb tides (Fig. 2a, b). The largest flocks of terns were encountered at SeaGen North during peak flood tides (Fig. 2c), with aggregations frequently exceeding 50 birds (Fig. 2a). On average, tern numbers observed foraging at the SeaGen North site during peak flood were three times as many as those foraging at either of the two natural wake sites (Fig. 2c). Because of high overdispersion and zero-inflation in the datasets, a hurdle-model was used to divide statistical analysis into presence-absence and count components^38^. In summary, the mean probability of encountering terns and number of terns if encountered per minute differed significantly among the wake features (Table 1). There were significant variations in probabilities of encountering terns and numbers of terns if encountered (Fig. 2a, b) across tidal states at most locations (an exception to this was SeaGen South).

**Table 1:** General-additive mixed effect model (GAMM) outputs with significance in both probabilities and numbers of terns among sites and within sites across tides

*Probability of encountering terns per minute*		
Among sites	*F*_3,1770_ = 109.8	*p* < 0.01
Across tides in SeaGen North	*F*_4,1769_ = 308.41	*p* < 0.01
Across tides in SeaGen South	*F*_4,1769_ = 1.60	*p* = 0.02
Across tides in Routen Wheel	*F*_4,1769_ = 5.64	*p* < 0.01
Across tides in Walter’s Rock	*F*_4,1769_ = 17.55	*p* < 0.01
***Number of terns per minute if encountered***		
Among sites	*F*_3,789_ = 33.69	*p* < 0.01
Across tides in SeaGen North	*F*_4,788_ = 34.28	*p* < 0.01
Across tides in SeaGen South	*F*_4,788_ = 0.00	*p* = 0.88
Across tides in Routen Wheel	*F*_4,788_ = 10.28	*p* < 0.01
Across tides in Walter’s Rock	*F*_4,788_ = 13.51	*p* < 0.01

The significane levels are *p* < 0.01

### Tern foraging in relation to man-made wake

**Fig. 3:**
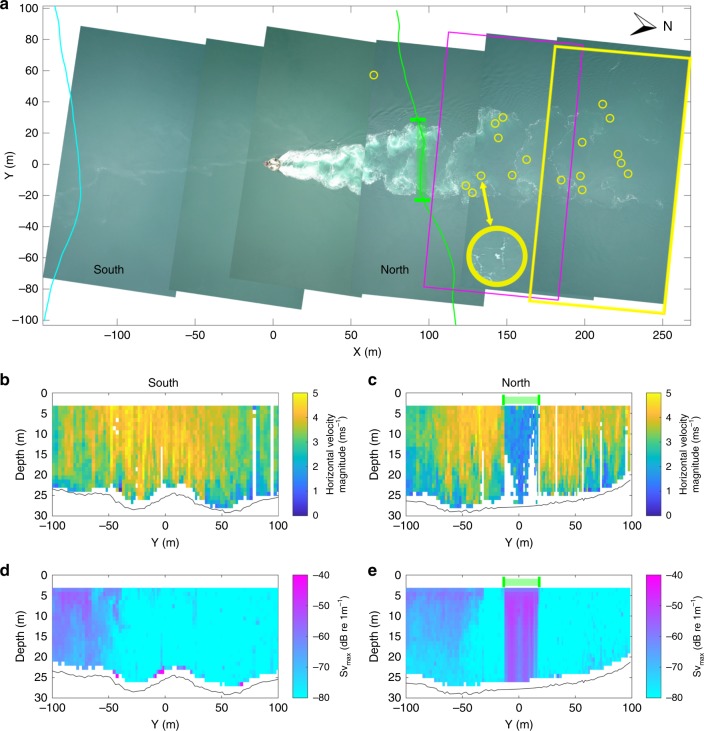
Tern distribution during peak flood tide in relation to SeaGen’s wake structure. **a** Georeferenced composite panoramic image from unmanned aerial vehicle (UAV) transect survey with terns identified (yellow circles—one enlarged for clarity). The orientation of the *x*-axis is 349 degrees. Magenta and yellow boxes indicate tracking regions shown in Fig. 4. **b**, **c** Horizontal velocity magnitude (ms^−1^) profile from the southern (cyan) and northern (green) ADCP transect, respectively. **d**, **e** Maximum acoustic backscatter (dB re 1 m^−1^) profile from the southern and northern ADCP transect, respectively. The North transects show a clear water column velocity deficit (**c**) and backscatter (an indicator for macro-turbulence) signature (**e**) in the area of the flood wake (*Y* = −20 to 20 m)

Overall, the probability and size of tern aggregations was highest at the man-made structure (SeaGen North), triggering a fine-scale investigation of its wake dynamics. Unmanned aerial vehicle (UAV) transects above SeaGen over several tidal cycles visualised the dynamic vortex shedding of the wake and the exact spatial extent of tern foraging, thereby overcoming the oblique angle of the vantage point observer. Consistent with the vantage point surveys, these transects recorded that terns focused their foraging activity almost exclusively over the flood wake (SeaGen North; Fig. 3a). The lee wake vortices showed the distinct and predictable pattern consistent with a von Kármán vortex street, with a surface-tracked eddy shedding frequency of 10–14 min^−1^.

To assess vertical wake effects throughout the water column, vessel-mounted acoustic doppler current profiler (ADCP) transects were run either side of the SeaGen foundation throughout a flood-ebb tidal cycle. The upstream near-laminar flow exceeding 5 ms^−1^ experiences a clear velocity deficit downstream in the midline of the structure throughout the water column with a cross-stream extent of 45 m at ~100 m downstream of SeaGen (Fig. 3b, c). The corresponding signature of elevated acoustic backscatter, an indicator for macro-turbulence^39^, visible in the downstream wake (Fig. 3e—compared to the upstream flow, Fig. 3d) is most likely dominated by entrained bubbles^40^, and to a lesser extent, sediment re-suspension^41^ and perhaps fish^42,43^. Bounded by the sea surface and seafloor, the backscatter signature from the wake of the structure is distinct from adjacent water. This provides evidence that the turbulent eddies within the flow are powerful enough to up-and down-well submerged material throughout the entire water column. While extreme water column scattering from bubbles and sediment precludes the acoustic extraction of fish targets from turbulence, the wake likely has the potential to act as a prey “conveyor belt” for surface foragers.

**Fig. 4:**
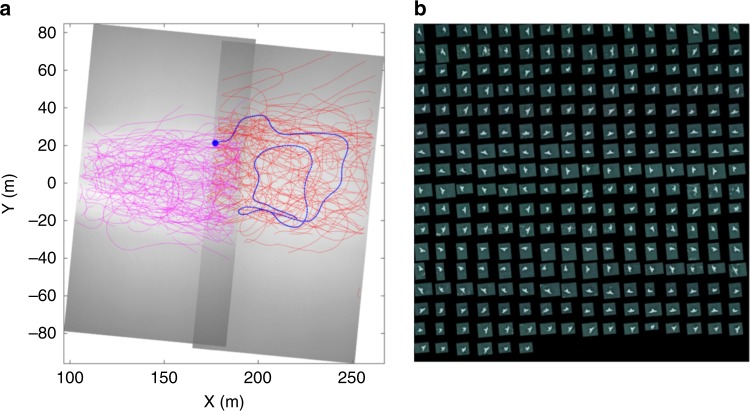
Tern flight trajectories recorded during peak flood tide in relation to SeaGen’s wake structure. **a** Georeferenced trajectories overlaid on time-average video images showing brighter region of foam/suspended material in wake. All trajectories of over 2 s duration are shown from recording periods of 140 s (red, 136 in total) and 125 s (magenta, 196 in total). **b** Sequence of images of an individual tern as it follows the trajectory indicated in blue in a (dot indicates start). Only every fourth image (0.16 s time interval) is shown for clarity in row-wise order starting at the top-left of the panel

Applying machine learning algorithms to distinguish terns from other moving targets (e.g., foam), flight trajectories recorded over the wake region (Fig. 4a) showed a high degree of in-flight sinuosity, typical for area-restricted search behaviour in response to increased prey intake rate/profitability (characterised by decreased flight speeds and frequent turning^2^, Fig. 4b). The terns forage almost exclusively over the vortex street with mostly transit flights to and from the colony outside of this central region.

### Particle flux corresponds with tern foraging patterns

Finally, the persistent use of the SeaGen (North) wake by the terns limited to the flood tidal cycle was explored using a hydrodynamic model coupled to an ecological module. Passive particles as a proxy for small prey organisms were released from the Irish Sea, outside the entrance of the Lough at the beginning of a flood tide (Fig. 1b). The flux of incoming potential prey items to SeaGen’s flood wake originates 70 min upstream from outside the Lough, corresponding with the rise in tern sightings ~60 min post low water slack.

## Discussion

To our knowledge, this is the first study to link indirect physical interactions (a downstream wake) of a renewable energy structure with top predators, highlighting the hitherto overlooked ecological implications of localised physical forcing around man-made structures. While top predator use of anthropogenic structures has been observed elsewhere^44,45^, distinct mechanisms may be in place to explain such associations. Namely, (1) natural reefing can increase fish biomass^46^, (2) fish can seek flow refuge in the immediate lee of a structure^34^ and (3) downstream wake effects can make incoming prey available near the surface through displacement^35,47^. The latter mechanism is currently the least explored in a natural setting despite its importance in high-flow environments, highlighting the relevance of our findings. While natural bathymetric features and associated patterns of shear lines and wake effects have been shown to attract top predators^8^, the man-made wake in this study promoted the most persistent and intense foraging aggregations of terns among all wake features investigated. While we did not assess prey vertical distribution, turbulent vertical velocity fluctuations within the wake were greater than 0.5 ms^−1^ (Supplementary Fig. 1), exceeding swimming performance of typical piscivorous tern prey items^13^ (e.g., sandeel^48^ in the order of 0.2 ms^−1^ or sprat/herring^49^ in the order of 0.4 ms^−1^) and may have the potential to displace prey. Therefore, our future studies will focus on assessing prey distribution and availability within both the inflows and wakes under different tidal states.

With the intensification of man-made structures in coastal seas, new synergies between these and marine predators are likely. Our findings demonstrate that wake features, predictable in time and space, persistently attract top predators at highly localised scales. We also provide the first empirical evidence that localised hydrodynamic forcing attributable to an anthropogenic structure can present a mechanism to promote a foraging hotspot, where predator aggregations exceed those at adjacent natural wake features. A broader perspective on the ecological implications of offshore installations is critical^23^ and requires the identification of such localised physical processes underlying top predator hotspot formation. For seabirds, there is concern that the introduction of renewable energy devices could lead to avoidance, thereby negatively impacting on energy expenditure^50^. Likewise, it has been suggested that hydrodynamic forces around hard structures could modify prey availability, thereby increasing a seabird’s rate of energy acquisition^51^. While our findings suggest that terns exploit the flood wake of a device, an overall ecological (population-level) benefit through increased individual energy acquisition can only be determined when accounting for parameters relating to, e.g., foraging success, prey profitability, and breeding performance^51,52^.

In the expanding renewable energy sector (e.g., > 4000 offshore wind turbines in Europe^53^), monopile foundations similar to the SeaGen design present the most common substructure (66%^53^) and lead to comparable wake vortices^25,27,54^. However, even submerged tidal turbines, and more so arrays, placed in unsteady flows will change the local hydrodynamic regime, including wake effects^26,55^ and more empirical data are required to predict changes in hydrodynamics and foraging habitat.

With SeaGen being decommissioned, its removal will undoubtedly change the foraging aggregations observed here. The decommissioning process, often requiring the complete removal of an aging structure^56^, is currently being re-considered globally by evidence of potential ecological benefits through artificial reef effects^57^ and increased fish biomass^46,58^ if parts remain in the sea. However, there is equal concern about the possible ecological impacts of artificial structures on marine vertebrates^59^ and in terms of their benthic footprint^60,61^. Renewable energy installations show some ecological synergies to oil-and gas platforms^44,60,62^ and could become an important contributor to the foreseen ʻdecommissioning crisis^63^ʼ if not addressed in a timely manner. Therefore, when designing the decommissioning removal scope of devices, a case-by-case determination of the ecological benefits or disadvantages of seemingly obsolete installations is required^64^.

## Methods

### Study site

All wake features investigated are situated in the Narrows, a tidal channel linking Strangford Lough, Northern Ireland, UK, with the Irish Sea (Fig. 1). The three sites investigated were (1) Walter’s Rock (54° 22.992’N, 5° 33.504’W), an island located on the periphery of the main channel, generating local upwelling and shear lines extending both into the channel and the near-shore shallows; (2) SeaGen (54° 22.122’N, 5° 32.766’W), located in the mid-channel experiencing the highest current magnitudes^39^ and (3) the Routen Wheel (54° 21.698’N, 5° 32.476’W), turbulent whirlpool structures that are generated from a shallow pinnacle (5 m depth) surrounded by 20 m deep waters. Here, the asymmetrical bathymetry of the channel promotes a more intense turbulence field at the surface during the ebb tide. While all three wake features differ in composition, they all predictably create local zones of extreme turbulent flow structures and tern feeding flocks had been observed at all three features prior to the study. With various tern (*Sterna sandvicensis, S. hirundo, S. paradisaea*) colonies located across Strangford Lough, Swan island presents the nearest colony to any of these wake features (Fig. 1). Sandwich terns are most abundant with 776 AONs (Apparently Occupied Nests, which equates to the number of breeding pairs), followed by common (340 AONs) and Artic (193 AONs) terns, respectively (pers. comm. Hugh Thurgate, National Trust, Strangford Lough head ranger).

### Data collection and analysis

A vantage point study was designed to collect count data of terns over the wake features between 18th July 2018 and 12th August 2018. Vantage points were located on the shore with a 200 m–1 km distance from each feature and covered an area of ~0.05 km² for each site to assess bird numbers associating with each localised wake feature. Observations covered all tidal states over a spring and neap tidal cycle. Using binoculars (Opticron Verano BGA HD and Nikon Monarch 10 × 42), counts of hovering or diving birds deemed foraging were completed every 2nd/3rd min for 15 min with a 5 min rest period to avoid observer fatigue (mean survey period across sites = 129 min, SD = 41 min). Number of surveys varied minimally per site, with Walter’s Rock (*n* = 9), SeaGen (*n* = 13) and Routen Wheel (*n* = 11) with a total observation time of 23.38 h, 25.26 h and 22.14 h, respectively. A general-additive mixed effect model (GAMM) was performed to quantify variances in the probability of encountering terns and the number of terns if encountered among tidal states and locations. A binomial model was used for the probability of encountering terns, and a negative binomial was used for the number of terns if encountered. Location was used as a categorical explanatory variable. Tidal state (hours after high water) was used as a continuous and non-linear explanatory variable. The number of knots was constrained to six to avoid over-fitting. Tidal state was also modelled as an interaction with location to account for differences in patterns among locations. An AR1 structure was used to account for temporal autocorrelation in model residuals within locations. Model parameters were used to predict variations in the probability of encountering terns and the number of terns if encountered across different locations and tidal states. Differences in probabilities and numbers across locations and tidal states were tested for significance (*p* < 0.05) using *F*-tests. Models were performed in the mgcv packages in R Statistics^65^.

### UAV surveys

To record fine-scale foraging behaviour in relation to the wakes, UAV surveys were performed from the nearest accessible shore location to each feature using a DJI Mavic Pro quadcopter recording 4 K video at 25 fps. The UAV was flown manually using the DJI Go v4.0 application. In order to comply with best practices^66^ and minimise potential disturbance, the vertical ascent of the UAV was made at 200 m distance from the foraging aggregations and sampling was performed at a height of 120 m above-surface level, as measured by the on-board altimeter. Missions included transects across SeaGen, as well as hovering (holding station with a vertically downward-facing camera) over the flood wake of SeaGen to capture seabird flight tracks over time. Surveys reported here were conducted on 11 July 2018 during a flood tidal cycle (07:30 h – 08:30 h GMT) with a total flight time of 41 min. All missions were completed in accordance with local regulations and flown by the same qualified (UK Civil Aviation Authority) pilot. The UAV camera was calibrated in the lab and video sequences post-processed using MATLAB (R2017b; Mathworks). Georeferenced composite panoramic images captured the distribution of terns up-and downstream of SeaGen. Machine learning approaches were used to identify, count and track terns over SeaGen’s flood wake. Briefly, moving objects were detected using frame-to-frame differencing, segmentation and then filtered by size to remove sun-glint speckles and large foam patches. Images of potential targets were then passed through a trained “Bag of Features” classifier before using Kalman filters to compile tracks of those targets identified as terns only. The classifier was trained using 806 manually identified images each of foam and terns, with an average accuracy of 93% when applied to a validation set of 3764 images.

### Acoustic doppler current profiler (ADCP) surveys

Vessel-mounted ADCP transects were performed on 13 Aug 2018 using a pole-mounted (1.15 m depth) RDI Workhorse Monitor broadband ADCP (600 kHz) in bottom-tracking mode with a vertical bin size of 1 m. All data was acquired using VMDas software (v. 1.46; RD Instruments, Inc.) and post-processed in WinADCP (v. 1.14; RD Instruments, Inc.). True current velocities were computed by subtraction of the bottom-tracked boat velocity. To quantify the acoustic scattering in the water column as a metric for macro-turbulence^39^, volume-backscattering strength (Sv in decibels, dB) was calculated across a maximum of 40 bins from the ADCP’s recorded raw echo intensity data using a working version of the sonar equation as originally described in Deines^67^ and updated by Mullison^68^. The backscatter equation accounts for two-way transmission loss, time-varying gain, water absorption, and uses an instrument- and beam-specific RSSI scaling factor to convert counts to decibels. This makes it a more robust measure of scattering compared to raw echo intensity, which can be more readily extracted from the ADCP. Sv was calculated for each bin along each of the four beams of the ADCP. For each range bin, the maximum of the four beams (Sv_max_) was taken to create depth profiles of the maximum level of scattering across the water column. In high-flow environments, high values of acoustic scattering are dominated by enhanced surface bubble entrainment and sediment re-suspension^22,41,69^.

### Hydrodynamic modelling

The Strangford Lough hydrodynamic model developed using MIKE21 modelling software (DHI Water and Environment software package: www.dhisoftware.com)^70^ was used to simulate particle movement in the Narrows. In short, the model uses a finite volume method by solving a depth averaged shallow water approximation. Full details of the model setup can be found in Kregting and Elsäßer^70^. The Strangford Lough model was coupled to a particle tracking module that incorporates advection and dispersion resolved using the Langevin equation. For horizonal movement, in the absence of any dispersion (horizontal or vertical) information, the scaled eddy viscosity was used with the software recommended constant value of 1.0. For the vertical dispersion, a constant dispersion value of 0.01 m^2^ per second was used. Changes in flow velocity throughout the water column were calculated based on the bed friction velocity, a parameter calculated directly in the hydrodynamic model. Passive particles as proxy for microscopic or small organisms were released from the Irish Sea at a depth of 10 m, approximately half the water column height (Fig. 1). A trickle release approach was adopted where 200 particles were released every 5 min timestep on the flood tide only and the time taken from release to the time taken to reach SeaGen was noted.

### Reporting summary

Further information on experimental design is available in the Nature Research Reporting Summary linked to this article.

## Supplementary information

Reporting Summary

Supplementary Information

## Data Availability

The dataset used to generate the main result shown in Fig. 2 is available online at 10.6084/m9.figshare.7732514.v1^71^. All other data generated and analysed during the current study are available from the corresponding author on reasonable request.
