# Supplementary material for: Localised anthropogenic wake generates a predictable foraging hotspot for top predators
Source: Commun Biol. 2019 Apr 4;2:123. doi: 10.1038/s42003-019-0364-z (PMC6449372; doi:10.1038/s42003-019-0364-z)
Supplement: Supplementary file 1 — Reporting Summary [file 42003_2019_364_MOESM1_ESM.pdf]

nature research

Corresponding author(s): Lilian Lieber

Reporting Summary

Nature Research wishes to improve the reproducibility of the work that we publish. This form provides structure for consistency and transparency in reporting. For further information on Nature Research policies, see Authors & Referees and the Editorial Policy Checklist.

Statistical parameters

When statistical analyses are reported, confirm that the following items are present in the relevant location (e.g. figure legend, table legend, main text, or Methods section).

n/a	Confirmed	
☐	☒	The exact sample size (n) for each experimental group/condition, given as a discrete number and unit of measurement
☐	☒	An indication of whether measurements were taken from distinct samples or whether the same sample was measured repeatedly
☐	☒	The statistical test(s) used AND whether they are one- or two-sided<br>*Only common tests should be described solely by name; describe more complex techniques in the Methods section.*
☐	☒	A description of all covariates tested
☐	☒	A description of any assumptions or corrections, such as tests of normality and adjustment for multiple comparisons
☐	☒	A full description of the statistics including central tendency (e.g. means) or other basic estimates (e.g. regression coefficient) AND variation (e.g. standard deviation) or associated estimates of uncertainty (e.g. confidence intervals)
☐	☒	For null hypothesis testing, the test statistic (e.g. F, t, r) with confidence intervals, effect sizes, degrees of freedom and P value noted<br>*Give P values as exact values whenever suitable.*
☒	☐	For Bayesian analysis, information on the choice of priors and Markov chain Monte Carlo settings
☐	☒	For hierarchical and complex designs, identification of the appropriate level for tests and full reporting of outcomes
☒	☐	Estimates of effect sizes (e.g. Cohen's d, Pearson's r), indicating how they were calculated
☐	☒	Clearly defined error bars<br>*State explicitly what error bars represent (e.g. SD, SE, CI)*

Our web collection on statistics for biologists may be useful.

Software and code

Policy information about availability of computer code

Data collection	No software was used during data collection
Data analysis	Data analysis for the general-additive mixed effect model (GAMM) was performed in R. Custom code written in Matlab (R2017a) was used for the ADCP (Acoustic Doppler current profiler) data visualisation, the UAV (unmanned aerial vehicle) panoramic image composition, as well as for the bird detection and tracking.

For manuscripts utilizing custom algorithms or software that are central to the research but not yet described in published literature, software must be made available to editors/reviewers upon request. We strongly encourage code deposition in a community repository (e.g. GitHub). See the Nature Research guidelines for submitting code & software for further information.

Data

Policy information about availability of data

All manuscripts must include a data availability statement. This statement should provide the following information, where applicable:

- Accession codes, unique identifiers, or web links for publicly available datasets
- A list of figures that have associated raw data
- A description of any restrictions on data availability

Raw data used to produce the key result (Figure 2) are available from figshare and the DOI has been provided. Other data are available from the corresponding author upon reasonable request.

Field-specific reporting

Please select the best fit for your research. If you are not sure, read the appropriate sections before making your selection.

☐ Life sciences ☐ Behavioural & social sciences ☒ Ecological, evolutionary & environmental sciences

For a reference copy of the document with all sections, see nature.com/authors/policies/ReportingSummary-flat.pdf

Ecological, evolutionary & environmental sciences study design

All studies must disclose on these points even when the disclosure is negative.

Study description	The study aimed to assess the influence of both natural and man-made wakes (characterised by intense macro-turbulence) on the foraging patterns of seabirds restricted to shallow plunge-diving foraging techniques (tern species, Sterna sandvicensis, S. hirundo, S. paradisaea). This was done by comparing the number of foraging terns at three different wake features (two natural wake features and one man-made wake feature) using a combination of a vantage point survey design with UAV transects. The anthropogenic wake was further investigated using active acoustic measurements derived from an ADCP (Acoustic Doppler current profiler). Finally, a hydrodynamic model was run to assess particle transport timing to the wake features.
Research sample	Seabirds counted at the three wake features included three tern species present in the study area (Sterna hirundo, S. sandvicensis, S. paradisaea). The rationale to selecting the two natural wake features as a comparison was that they presented the most 'extreme' natural features creating turbulence most resembling the anthropologically-generated wake. Terns have been observed at all wake features prior to the study. While the vantage point seabird count data was collected over a month, the ADCP and UAV data were collected during a representative flood-ebb tidal cycle as the physical character of the wake primarily varies with tidal state.
Sampling strategy	The number of wake features considered in this study were chosen to provide directly comparable context to the anthropogenic wake feature. Seabird counts at each feature were conducted during different tidal states (ebb, flood, spring and neap tides; >3 weeks) to assess the relative use by terns of each of the features during varying hydrodynamic conditions.
Data collection	The number of seabirds deemed foraging over each of the features were counted using a vantage point survey methodology. JJ Waggitt and L Lieber conducted the shore surveys from neighboring shores (200-1km distance). All UAV transects were performed by WAM Nimmo-Smith (UK Civil Aviation Authority qualified pilot). The UAV take off locations were planned at a 200m distance from seabird foraging aggregations and flights performed 120m above-ground level to minimize potential disturbance to wildlife. The ADCP data were collected by L Lieber from a 10.5m long offshore-coded vessel (The Cuan Shore, Cuan Marine Services Ltd).
Timing and spatial scale	The vantage point surveys were conducted during the summer months (July-August) in 2018 when summer-breeding terns are present in the study area. The UAV transects were conducted on 11 July 2018 within a radius of 250m from the wake feature. The ADCP data were collected on 13th August 2018. Data was collected at local scales where vantage sites covered approximately 0.05km². The ADCP transects were run at a 100m distance from the anthropogenic structure to capture the near-field underwater properties (velocity and backscatter) of the wake.
Data exclusions	No data were excluded.
Reproducibility	A full description of the methodology used can be found in the Methods. All survey types (vantage point counts, UAV transects and ADCP transects) were replicated successfully to ensure reproducibility.
Randomization	The wake features selected for this study were not allocated randomly, but were chosen based on their physical properties and tern interactions. The rationale of this study was to investigate bird numbers at local scales (<1km) and therefore all data collection focused on the same spatial extent. However, vantage point surveys (time of day, day of the week) were random while maintaining sampling effort (number of surveys) equal across sites and tidal states. Most days, all three vantage points were rotated, thereby covering all tidal states at each site.
Blinding	Blinding was applied in so far that samplers had no prior knowledge of species location and composition across sites. Otherwise, blinding was not relevant or possible during this study.

Did the study involve field work? ☒ Yes ☐ No

Field work, collection and transport

Field conditions	All data was collected during sea state of 0–2 and a visibility of 6–10 km. No data was collected during heavy rainful as that would have impacted species determination.
Location	The study was performed in the Narrows tidal channel, located in Strangford Lough, Northern Ireland, UK (54.4830° N, 5.5830° W). The Narrows are approximately 8 km long with a minimum width of 1 km and depth varying between 30–60m in the mid-channel. The Narrows link Strangford Lough with the Irish Sea and experiences high current flows in excess of 5m/s.
Access and import/export	Correspondence with the local department of environment (DAERA) prior to the study confirmed that no permits for either the vantage point surveys, the ADCP data collection, nor the UAV transects was necessary. The UAV transects were performed according to UK Civil Aviation Authority regulations and with the consent of the landowner for take off and landing.
Disturbance	Observational data (e.g. vantage point surveys) did not cause any disturbances. No animals were sampled or approached and in order to minimize potential disturbance to foraging birds during the UAV transects, the take off and landing point of the UAV transects was chosen at a 200m distance from foraging birds and the UAV was flown at 120m above-sea level, exceeding common recommendations regarding best practices when using UAVs for wildlife studies.

Reporting for specific materials, systems and methods

Materials & experimental systems

n/a	Involved in the study	
☒	☐	Unique biological materials
☒	☐	Antibodies
☒	☐	Eukaryotic cell lines
☒	☐	Palaeontology
☐	☒	Animals and other organisms
☒	☐	Human research participants

Methods

n/a	Involved in the study	
☒	☐	ChIP-seq
☒	☐	Flow cytometry
☒	☐	MRI-based neuroimaging

Animals and other organisms

Policy information about studies involving animals; ARRIVE guidelines recommended for reporting animal research

Laboratory animals	The study did not involve laboratory animals.
Wild animals	All wild animal work in this study was observational and animals observed in this study (from a 200m-1k distance) included three tern species Sterna hirundo, S. sandvicensis, S. paradisaea). However, no animal was captured, sampled or transported.
Field-collected samples	The study did not involve samples collected from the field.
