# Supplementary material for: Localised anthropogenic wake generates a predictable foraging hotspot for top predators
Source: Commun Biol. 2019 Apr 4;2:123. doi: 10.1038/s42003-019-0364-z (PMC6449372; doi:10.1038/s42003-019-0364-z)
Supplement: Supplementary file 2 — Supplementary Information [file 42003_2019_364_MOESM2_ESM.pdf]

Supplementary Fig. 1

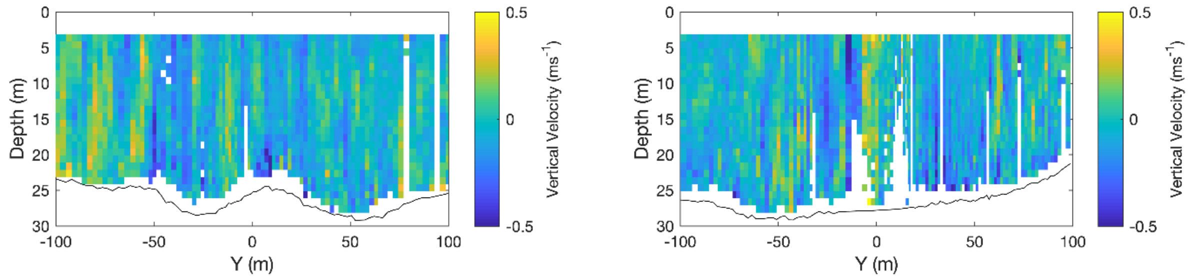

Supplementary Fig. 1: Vertical velocity (ms^{-1}) profile from the southern (left) and northern (right) ADCP transect, respectively.
